# Man or machine? Will the digital transition be able to automatize dietary intake data collection?

**DOI:** 10.1017/S1368980018003993

**Published:** 2019-05

**Authors:** Bent Egberg Mikkelsen

**Affiliations:** Aalborg University AC Meyersvænge 15 DK-2450 Copenhagen SV, DenmarkEmail: bemi@learning.aau.dk

Data collection in studies of dietary intake has traditionally relied on labour-intensive methods requiring inputs from the individual studied. As a result, such studies are costly and time-consuming. But the emergence of digital technologies has sparked a new interest in methods and approaches that can be used for automated collection and processing of data on food intake. New sensor technologies, smarter interfaces and progress in artificial intelligence (AI) research seem to be boosting the field and bringing the field forward.

Information and communication technology (ICT) assistance for dietary data collection has been available for a number of years, including both web- and application (app)-based solutions where participants can enter dietary information directly interfaced to a database. Most replace a simple paper-and-pen approach with a keyboard or a smart screen but do not radically change the workload required by the participant. What the digital transition offers is the smart application of new sensors and devices that can ‘think and sense’ themselves and thus remove partly or fully the workload and responsibility for data acquisition from the participant. As such, we can distinguish between (i) more simple ICT-assisted self-reporting dietary data acquisition and (ii) automated or semi-automated dietary data acquisition. The first type covers ICT-based solutions where paper-based methods are replaced with screen-based ones, examples of which have been reported^(^ ^1^ ^,^ ^2^ ^)^. The second type covers methods that automatize the data entry by using sensors and/or AI to reduce workload, such as the chest-worn micro camera computer called eButton^(^ ^3^ ^)^ and the mini-suitcase format Dietary Intake Monitoring System (DIMS) used for bedside food intake monitoring in hospitals^(^ ^4^ ^)^.

Scholars are testing the reach of new technologies, sensors and devices for the study of food intake in experimental laboratory settings such as the Foodscape lab at Aalborg University^(^ ^5^ ^)^, the Fake Food Buffet lab at the Technical University of Zürich^(^ ^6^ ^)^ and the Restaurant of the Future at Wageningen University^(^ ^7^ ^)^. Along with the progress made in the Technology Assisted Dietary Assessment (TADA) project^(^ ^8^ ^,^ ^7^ ^)^, the efforts to develop automatic and semi-automatic approaches have brought about devices such as the Mandometer Technology^(^ ^9^ ^)^, the eButton^(^ ^10^ ^)^, the DIMS^(^ ^11^ ^)^ and the Splendid approach^(^ ^12^ ^)^.

This issue of *Public Health Nutrition* presents eight studies on semi-automated dietary data acquisition and ICT-assisted self-reported dietary data acquisition. They fall in two groups: (i) applications for use in research settings and (ii) applications meant to be used by consumers in real-life food environments. They deal with important aspects including proof of principle over validity, reliability, user-friendliness and feasibility.

## Applications for use in research settings

Four of the papers deal with studies carried out in lab or health-care settings and explore applications using advanced imaging and language processing relying on AI.

Jia *et al.* ^(^ ^13^ ^)^ set out to develop an AI-based algorithm which can automatically detect food items from images acquired by an egocentric wearable camera for dietary assessment, the chest-worn eButton. The eButton categorizes pictures into food-related ones and uses AI to create tags of the pictures. The study tested the algorithm on two data sets, and results suggest that the approach has the potential to automatically identify foods from low-quality, wearable camera-acquired images with reasonable accuracy. That in turn seems to be able to reduce both the burden of data processing and privacy concerns. However, the estimation of volume/weight is an issue if results are to be translated into nutritional values.

The reliability and validity of the eButton are studied in the paper by Beltran *et al*.^(^ ^14^ ^)^, which examines the use of a digital screen-based wire mesh procedure to determine food amounts. The eButton wire mesh is a method that attempts to solve the issue of estimation of portion sizes. The estimation is done on screen images of foods by an assessor who fits a mesh from a mesh library on to each food. The authors found good reliability and validity comparing size estimations from two types of practitioners, although a difference between dietitians and engineers was found. The mesh approach seems to be promising to estimate amounts, and it could be anticipated that training a machine to perform this is a possible way forward to automatize the process.

The study by Ofei *et al.* ^(^ ^15^ ^)^ examines the validity of the DIMS technology, developed for monitoring of food intake among hospital patients, by comparing it with the weighed food method. The DIMS has previously been shown to reduce workload and offers an easy way to determine pre- and post-serving portion sizes and type estimation, although the post-serving conditions have been more challenging since leftovers are often mixed together^(^ ^16^ ^)^. However, the results of the study showed a significant correlation between the two methods and suggest that the DIMS is a valid, novel, easy-to-use alternative for monitoring of dietary intake in hospital settings.

Mezgec *et al.* ^(^ ^17^ ^)^ present a study using a two-step process to estimate food intake. The authors use a deep learning approach to picture-recognition of images representing choices from the ‘fake food buffet’ to tag images, and a subsequent language processing approach to match the tags with entries in a food composition database. The results of the study show that the accuracy of the deep learning model trained to tag fake-food images as well as of the matching of the resulting words with the food composition database was satisfactory. The study shows that the feasibility of semi-automatically creating a description of food items that links it to, for instance, the FoodEx2 language classification system offered by the European Food Safety Authority. This means that a direct link can be created to relevant food composition databases.

## Applications for use in consumer settings

Four of the papers relate to studies of smartphone-based applications. Two of them deal with approaches that use the camera to collect pictures for further processing in a research context, whereas the two other studies examine applications meant for consumer-targeted nutrition information that display information on foods sourced from databases.

Yang *et al.* ^(^ ^18^ ^)^ present a picture-based approach and explore the utility of a new smartphone-based imaging method that works without a fiducial marker. Instead it applies virtual reality, automated training and a standard food unit to estimate the portion size. The imaging approach estimates food volumes that need to be converted to amounts post-process. The method was found to be valid and a training sequence improved estimation accuracy significantly.

The study by Prinz *et al.* ^(^ ^19^ ^)^ examines the validity of a mobile camera by comparing it with weighed methods, and also evaluates user satisfaction. The results show a significant correlation between the two methods in estimation of energy, macronutrient and fibre intakes, although the authors found a systematic bias with increasing levels of intake. With respect to user friendliness, a large majority of participants were satisfied with the photo-based method and had few technical challenges.

Braz and Baena de Moraes Lopes’ study^(^ ^20^ ^)^ aims to verify the reliability of information, the sources of information used and the user opinions of sixteen free mobile apps with nutritional information available in Brazil. They found that the accuracy, in the case of energy, ranged from 0 to ~57 %. Not surprisingly the authors concluded that the apps are not useful for nutritional guidance.

Food composition is increasingly available for brands and calculating nutritional value through barcodes and Universal Product Codes has become technically available, along with app-based solutions. Maringer *et al.* ^(^ ^21^ ^)^ study the option of getting in-shop access to nutritional values through a scanning approach. More specifically, they examine the quality of the results obtained from labelled food product databases using a barcode scanner to link the food to the database value. The results of the study showed that energy values could be retrieved in almost all cases. For nutrients, however, availability and accuracy varied greatly across the apps. Since open access to data about branded foods is increasing, this approach to converting food intake to energy intake is a promising avenue to explore further.

## Discussion

The papers all together clearly illustrate the width and the scope of current research efforts at the intersection of food and digital technology. They cover new applications of smartphones that can be used in a consumer environment as well as cutting-edge technologies and hardware. The studies cover important aspects of validity, reliability, convenience and feasibility.

First and foremost, imaging and language processing technologies are an important part of the solutions reported in this issue. The papers suggest that computer-based tools that help us make sense of how we see and how we speak about food are at the core of these scientific efforts. The task of dietary assessment can be described in simple terms as ‘measuring the type and amounts of a dietary record and linking it to an authoritative table that can return the energy and the nutrient content’. But in practice it represents a huge technological and scientific challenge.

Besides images and language, current research is investigating the use of proxies and indicators of intake in order to find specialized hardware solutions that can assist in specifying types and amounts of food. These include scales, jawbone motion sensors, chewing and swallowing audio sensors, fork motion sensors, hand movement sensors, spectrophotometry vision, electromyography vision and near-field communication^(^ ^16^ ^,^ ^22^ ^–^ ^27^ ^)^. These research directions can advance the science of automated dietary assessment assuming the necessary cross-disciplinarity across nutrition, food and ICT is established. At Aalborg University we have established a strategic interdepartmental cooperation called the Digital Foodscape Lab that aims to bridge the gap across studies of food and nutrition, mobile devices, communication, robotics and mediology. Such cooperation makes it possible to develop and test prototypes of applications in real-world environments under realistic conditions, with the assistance of both students and early career researchers, while keeping costs at an acceptable level.

A synthesis of the eight papers points in this issue to directions and priorities for future research needs, including:

∙ automatic or semi-automatic portion size estimation;
∙ correct estimation of leftovers and plate waste;
∙ creation of more direct links to foods for which nutritional contents are already known, especially since they might appear in a branded food composition table;
∙ better computer vision technologies and algorithms for classifying foods;
∙ smarter ways to directly monitor foods purchased at the point of sale;
∙ research on how to address privacy concerns and full compliance with the General Data Protection Regulation^(^ ^28^ ^)^; and
∙ closer cooperation internationally across research groups.

Digital solutions for better health care is a trending topic at regional, national and EU levels. The European Strategic Forum on Research Infrastructures (ESFRI) is currently addressing some of the challenges specifically related to food, and food, nutrition and health is expected to be a topic in the next ESFRI roadmap. One of the contexts that might provide infrastructure to facilitate such work is the Food, Nutrition and Health Research Infrastructure (FNHRI) suggested by the EU Richfields design study^(^ ^29^ ^)^. The FNHRI will try to improve access to data and strengthen the infrastructure between groups and labs working with digital-assisted solutions.
